# Two Sites of Obstruction with Gallstones: A Case Report of Bouveret Syndrome with a Concurrent Biliary Ileus

**DOI:** 10.1155/2023/9664165

**Published:** 2023-07-14

**Authors:** Eric Bergeron, Maude Pichette

**Affiliations:** Department of Surgery, Charles-Le Moyne Hospital, Greenfield Park, QC, Canada

## Abstract

Bouveret syndrome is a gastric outlet obstruction, and biliary ileus is an obstruction of the small bowel, and both are caused by a gallstone that escaped the gallbladder through a bilio-enteric fistula. The concurrent occurrence of obstruction at both sites is encountered very rarely, and only two such cases associated with Bouveret syndrome were reported before. We now present a case involving a 78-year-old female with simultaneous obstruction at both the duodenum and jejunum. The literature is reviewed to evaluate the incidence of such a situation and to discuss the management of the case.

## 1. Introduction

Bouveret syndrome specifically involves an obstruction of the stomach secondary to an impacted gallstone from a bilio-enteric fistula [1], usually formed due to an abnormal cholecystoduodenal communication [2]. Biliary ileus, also known as gallstone ileus, refers to an impacted gallstone within the lumen of the bowel causing obstruction [2–4]. Multiple stones may be retrieved in the digestive tract [5–7], but recurrence is uncommon [2, 3, 8]. Nevertheless, simultaneous dual-site obstruction is very rare [9–12], with only two earlier reported cases involving Bouveret syndrome in the literature [7, 13]. In the present report, we describe an interesting case of Bouveret syndrome causing gastric outlet obstruction with a simultaneous obstructive gallstone in the jejunum.

## 2. Case Presentation

A 78-year-old patient presented at the emergency department after a two-day history of vomiting, abdominal pain, and distension. She was diagnosed 16 years before to have antiphospholipid syndrome when she suffered from thrombophlebitis, pulmonary embolism, and hypertension. She was on apixaban, perindopril, dexlansoprazole, and citalopram. On physical examination, she was afebrile, with a lightly distended abdomen, but without defense or rebound tenderness.

**Figure 1:**
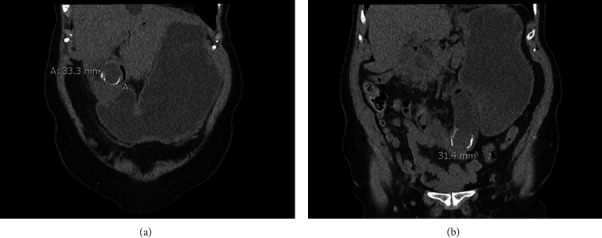
(a) Abdominal CT scan showing a 33-mm stone causing gastric outlet occlusion. (b) A 31-mm stone causing obstruction of the proximal jejunum.

An abdominal computed tomography (CT scan) was ordered and showed a distended stomach, duodenum, and proximal jejunum (Figure 1). An obstructive 31-mm stone was observed at the proximal jejunum. Another 33-mm stone was found under the liver at the gallbladder fossa. The location of the proximal stone, either within the gallbladder or in the pyloroduodenal region, could not be determined precisely. A small amount of aerobilia was demonstrated.

The patient was evaluated by an internist, who suggested waiting 48 hours to undergo surgery while apixaban is discontinued. Since the patient was stable and neither toxic nor in peritonitis, a decision to postpone the surgery was made. This delay also permitted rehydration and stomach decompression of the patient with a nasogastric tube.

On the third day post-admission, the patient was still stable and was taken to the operating room. A right subcostal approach was undertaken. There was a high degree of inflammation and adhesions in the subhepatic area. A large stone was palpated in the first part of the duodenum. The second stone was found in the middle of the small bowel, appearing farther than the location described on the CT scan. The stone was firmly impacted to the bowel wall, and a short resection was necessary to extract the stone. Then, the whole bowel was inspected, and no other stone was palpated. Thereafter, a distal gastrotomy was carried out, and the stone located in the first part of the duodenum was retrieved through the pylorus with some sponge forceps. The gastrotomy was then closed with a linear stapler.

The patient was kept with a nasogastric tube for the first four days. Diet was gradually resumed at this time. She was discharged on the eighth postoperative day. She was seen a month later following an uneventful recovery period. Six months later, there is still no evidence of recurrent gallstone-related problems.

## 3. Discussion

Gallstone ileus is a mechanical intestinal obstruction due to the impaction of gallstones within the lumen of the bowel [2]. Specifically, Bouveret syndrome involves obstruction of the stomach secondary to an impacted gallstone in the duodenum [1]. The gallstone escapes through a cholecystoduodenal fistula in the majority of cases [2]. Jejunum and ileum are the most common sites of obstruction [2, 4], whereas the stomach and duodenum may be involved in up to 14% of cases [2, 4, 13].

Gallstone ileus is estimated to occur in less than 0.5% of patients with gallstones and is responsible for less than 5% of intestinal obstruction [2]. However, 25% of intestinal obstructions in patients older than 65 years are attributable to gallstones [2, 3, 8, 14, 15]. Multiple stones may be retrieved in the digestive tract [5–7], and recurrence is reported between 2% and 8% [2, 3, 8]. Nearly, half of the episodes of recurrence occur within one month [2, 3, 6], and recurrence in the immediate postoperative period has also been reported [4, 8, 14–16]. The presence of two concurrent sites of obstruction is, however, a very rare situation [9–12].

The present case is a typical Bouveret syndrome with an obstructing stone in the first part of the duodenum [1]. An abdominal CT scan initially identified two stones, one seen clearly in the proximal jejunum, causing bowel obstruction (Figure 1). The location of the other stone could not be precisely defined on this examination, but we were convinced that it was already in the duodenum. Even though endoscopic removal could have been attempted, it was not considered as there was already an indication for surgical exploration. Moreover, 91% of patients would need surgery despite endoscopic treatment [17, 18]. Concerning the stone in the jejunum, it certainly moved more distally while awaiting surgery, and such movement was previously reported to occur [6, 12, 14, 19]. The standard management of the gallstone ileus is enterolithotomy and stone extraction [2–5, 15, 19, 20], or with resection of irreversibly damaged parts of the small bowel [4, 7, 8, 15, 20], as in the present case. The stone in the duodenum of our patient has been managed following standard procedures with the extraction of the stone through a gastrotomy [1, 13, 17].

Cholecystectomy and closure of the duodenal fistula were not planned as in one-stage surgery [6]. The procedure would have been time-consuming and technically challenging [2, 4, 5], considering the inflammation and the encountered adhesions. Besides, an absence of any retained gallstone in the gallbladder also advocated against the option of cholecystectomy and fistula closure [20]. Laparoscopy, although feasible but with high rates of conversion [18] was also not contemplated in this potentially difficult case. Bowel resection, which was necessary in the present patient, was attributable to the planned delay and is known to be associated with higher complication rate and mortality [20]. This patient also had to undergo an additional procedure, the gastrostomy, which further increased the magnitude of the urgent surgical intervention [3]. A second-stage cholecystectomy will probably be unnecessary [6, 8, 14, 18], considering that the majority of the bilio-enteric fistulas close spontaneously [2, 3, 8, 14, 18], particularly if no stones are remaining in the gallbladder [3, 4, 8, 18]. In emergency situations, the main goal of therapy must remain the relief of small bowel obstruction [3, 6]. Physicians must be aware of different surgical options [17] in these unusual, but not so rare situations [2, 3, 8, 14, 15].

Only two cases involving Bouveret syndrome associated with concurrent obstructive gallstones along the digestive tract were reported earlier in the literature [7, 13], with the first one in the sigmoid part of the colon [13] and the second one in the jejunum [7]. During surgical exploration, it is of major importance to palpate the digestive tract to rule out missed gallstones that could cause subsequent intestinal obstruction [2, 5, 8, 10–12], since stones can be multiple [6, 7, 9–13, 21], migrate [8, 14, 19, 22], or be unidentified on imaging [21, 23, 24]. Even though CT scan has a better diagnostic yield than plain abdominal X-ray [1, 21, 24] with a sensitivity of 93% [21, 23], it certainly cannot be a substitute for a thorough inspection of the bowel, that is, an essential part, of the treatment of gallstone ileus [2].

## 4. Conclusions

In summary, this is the third reported case of Bouveret syndrome associated with a concurrent site of intestinal obstruction caused by gallstone. Gallstone ileus is a situation that should be considered not so uncommon in the elderly population. Multiple stones should be carefully searched for during surgical intervention. Definitive treatment must be individualized but emergency intervention must be directed towards the correction of mechanical obstruction.
